# How Monkeys Are Captured

**Published:** 1890-09

**Authors:** 


### HOW MONKEYS ARE CAPTURED.

Most all monkeys which one sees in the United States come from Gorgona, a little village which is situated a short distance from the Panama Railroad. The inhabitants of this district are mostly native negroes, for no white man could bear the climate without drinking plenty of whiskey and almost continually swallowing quinine. The whole region is marshy and covered with extremely profuse tropical vegetation. At night there arises a thick vapor laden with fever, which hangs over the woods like a cloud.

This region of wood is the prairie of the monkeys. They travel in troops around the woods, led by an older monkey. When the people receive the information that the "traveling monkey troops" are near the village, they repair to the woods in crowds in chase of them. Their plan is very simple. They cut a hole in a cocoanut large enough for a monkey's paw. The nut is then hollowed out and a piece of sugar is placed in it. A piece of string is then fastened to it, and it is placed in the road of the approaching monkeys. It is known monkeys are very inquisitive animals. Soon enough they see the "lonesome" cocoanut in the grass and hurry to examine it thoroughly. It is a curious sight to see how they climb from the trees, chattering, to take a good view of the concern.

It does not take them long to find out that the inner part contains a piece of sugar. One of the boldest and greediest sticks a paw into the nut to get the sugar and grasps it as tightly as he can. But his fist is so large that he cannot draw it out of the hole again with the sugar, which he holds fast to, cost what it may. The negroes now pull the string until nut and monkey arrive in the vicinity of their ambuscade. In the meantime the other monkeys wonder what is the matter with their comrade. They hurry to see where he is being pulled to with his paw, in the cocoanut. They crowd around him, chattering and gesticulating to their hearts' content.

Now the great moment has come. The negroes have a large net ready, and they spread it out over the unsuspecting monkeys, and before they know it they are prisoners. They are sold to the employes of the Panama Railroad, and reach the North American market through commercial dealers.

